# A Traditional Mediterranean Diet Effectively Reduces Inflammation and Improves Cardiovascular Health

**DOI:** 10.3390/nu11081842

**Published:** 2019-08-09

**Authors:** Cristina Razquin, Miguel A. Martinez-Gonzalez

**Affiliations:** 1 Department of Preventive Medicine and Public Health, University of Navarra, 31009 Pamplona, Spain; 2 IdiSNA, Navarra Institute for Health Research, 31008 Pamplona, Spain; 3 CIBER Fisiopatología de la Obesidad y Nutrición (CIBERObn), Instituto de Salud Carlos III, 28029 Madrid, Spain; 4 Department of Nutrition, Harvard T.H. Chan School of Public Health, Boston, MA 02115, USA

Chrysohoou et al. fifteen years ago, showed in an elegant analysis nested within the ATTICA study [1] that a dietary score reflecting adherence to the traditional Mediterranean diet (MedDiet) was inversely associated with plasma biomarkers of low-grade inflammation. Specifically, participants in the highest tertile of adherence to the MedDiet presented 20% lower levels of highly-sensitive C reactive protein (hs-CRP), 17% lower levels of interleukin-6 (IL-6), and 14% lower white blood cell counts. This was an observational study that could be affected by residual confounding and other potential imperfections. However, another similar study, this time nested within the Nurses Cohort in the USA [2], assessed hs-CRP, IL-6, E-selectin, soluble intercellular cell adhesion molecule 1, and soluble vascular cell adhesion molecule 1 and found that better adherence to the MedDiet was also associated with a reduction in inflammatory biomarker concentrations, with relative reductions of 24% in hs-CRP, 16% in IL-6, and 13% in E-selectin concentrations [2]. These well conducted observational studies were subsequently confirmed by a randomized clinical trial (the pilot study of the PREDIMED (PREvención con DIeta MEDiterránea) trial) where we were able to show that an intervention with 2 MedDiets maintained during 3 months was able to reduce hs-CRP, IL-6 (in both cases) and adhesion molecules compared to a low-fat diet [3]. However, hs-CRP was reduced only when the MedDiet was supplemented with polyphenol-rich extra-virgin olive oil, but not with nuts.

Subsequently, the concordance of a good number of new observational studies and randomized clinical trials robustly and consistently showed that interventions with the MedDiet, better adherence to the MedDiet or use of extra-virgin olive oil could both effectively reduce the levels of biomarkers of low-grade inflammation implicated in the mechanisms leading to atherosclerotic disease [4,5]. An especially interesting finding was that reported by Medina–Remon et al. [6] in a substudy of 1139 high-risk participants nested within the PREDIMED randomized trial. We assessed the results of the intervention with polyphenol-rich MedDiets in these participants of the PREDIMED trial and found that urinary total polyphenols were associated with decreased inflammatory biomarkers, suggesting a dose-dependent anti-inflammatory effect of polyphenols, because participants in the highest tertile of changes in urinary total polyphenol excretion showed in parallel the highest reductions in plasma levels of inflammatory biomarkers [6].

In a recent study published in Nutrients, Sureda et al. [7] selected a random sample of the population of the Balearic Islands in Spain and assessed a score of adherence to the MedDiet using a previously described method [8]. They found that lower adherence to the MedDiet was directly associated with a worse profile of plasmatic inflammation markers. These results are interesting, because they used a highly sensitive score of adherence to the MedDiet [8], and they showed stratified results by sex and age strata and also assessed additional biomarkers, adiponectin, and leptin. They can provide further insights into the anti-inflammatory potential of the MedDiet.

The rich content in fruit and vegetable in the MedDiet contributes to its high anti-inflammatory potential as well as to other pleiotropic benefits provided by the polyphenols and other bioactive molecules present in fruits, vegetables, extra-virgin olive oil, whole grains, and wine. Interestingly, red wine and extra-virgin olive oil contain several bioactive polyphenols (hydroxytyrosol and tyrosol, oleocanthal, and resveratrol) with postulated anti-inflammatory properties. One or two decades ago, anti-atherogenic properties of olive oil were mostly attributed to its fatty acid composition, because it was an interesting source of monounsaturated fat (oleic acid). However, a good body of more recent evidence supports that bioactive polyphenols, only present in the extra-virgin variety of olive oil, may importantly account for its cardio-protective properties [9], specially related to reductions in low-grade inflammation [5].

It is also well known that nutrients do not act individually, but they have synergistic effects because of their combined intake. Specific polyphenols provided by the different foods typical of the MedDiet probably work in synergistic interactions. These interactions may explain why Sureda et al. [7] did not find any particular association for any of the individual components of the MedDiet score with inflammatory biomarkers, but they did find inverse associations between the overall MedDiet score and these biomarkers. Once more, the whole MedDiet is more than the sum of its parts [10].

An immense body of observational and experimental evidence supports that better adherence to the MedDiet is strongly associated with a reduction in the risk of major cardiovascular disease [11]. In fact, there is no such high accrual of evidence for any other dietary pattern as for the MedDiet.

It is well established that downstream biomarkers of inflammation such as IL-6 and hsCRP are associated with an increased risk of clinical cardiovascular end-points in observational studies, regardless of cholesterol levels. Therefore, the anti-inflammatory potential of the MedDiet derived from its high polyphenol content is likely to provide an interesting additional mechanistic explanation for the consistently observed findings in preventive cardiology, adding biological plausibility to support a causal effect of the MedDiet in reducing atherosclerotic disease. This is particularly supported by the results of the PREDIMED randomized clinical trial that go beyond mere observations [12,13]. The intervention conducted in the PREDIMED trial in 7447 participants [14] was focused on modifying the overall dietary pattern using a 14-item tool that captured a variety of targets for dietary modifications. In fact, the 14-item tool used in the PREDIMED trial to achieve these modifications was one of the three scores that appropriately captured the highest intake of polyphenol antioxidant content in a comparison among 21 MedDiet indexes [15].

A randomized clinical trial, the canakinumab anti-inflammatory thrombosis outcome study (CANTOS), published two years ago, was specifically designed to evaluate the inflammatory hypothesis of atherosclerosis (“proof-of-concept trial”) [16]. CANTOS reported the efficacy and safety of canakinumab, a monoclonal antibody that by inhibiting interleukin-1beta, reduces the levels of IL-6 and hs-CRP and exerts no effect on lipid levels. The treatment with 150 mg of canakinumab every three months significantly reduced major cardiovascular endpoints, including acute myocardial infarction, stroke, and cardiovascular death, obtaining a 15% relative reduction (95% confidence interval, 26% to 2%) in the risk of a major cardiovascular event compared to placebo. Reducing hs-CRP levels without any influence on the plasma lipid profile represents a splendid demonstration of the inflammatory-related mechanisms involved in cardiovascular disease. The cardiovascular benefit of canakinumab should only be attributed to its anti-inflammatory action. This finding strongly confirms the inflammatory hypothesis of atherosclerosis put forward by Ross two decades ago [17]. However, in the CANTOS trial, a higher risk of mortality associated with infections was found in those treated with canakinumab than in the placebo group. More importantly, the cost of treatment with canakinumab in the United States has been estimated at $200,000 per year, a figure that is unacceptably high from a public health perspective when considering the number needed to treat [18].

In this context, and given that the currently available evidence supports that the polyphenol-rich diets can have important anti-inflammatory actions, the dietary approach to combat inflammation presents all sort of advantages. At the end of the day, one hundred percent of the population is exposed to one diet or another, and promoting an anti-inflammatory dietary pattern may be perhaps the most effective, efficient, and sensible approach for preventive cardiology. In this line of thought, the ideal model for cardiovascular prevention, demonstrated beyond any other model, is the olive-oil rich traditional Mediterranean diet [11].
